# Abolishing User Fees in Africa

**DOI:** 10.1371/journal.pmed.1000008

**Published:** 2009-01-06

**Authors:** Valéry Ridde, Slim Haddad

## Abstract

Valéry Ridde and Slim Haddad discuss a new trial in Ghana in which households were randomized into a pre-payment scheme allowing free primary care or to a control group who paid user fees for health care.

## User Fees and Health Care Exclusion

In its 2008 annual report, the World Health Organization (WHO) urged countries to “resist the temptation to rely on user fees” [1, p. 26]. Indeed, the consensus in the scientific community is that user fees have harmful effects on health care use and household budgets, especially for the poorest [2]. Still, as the WHO observes, “…most of the world's health-care systems continue to rely on the most inequitable method for financing health-care services: out-of-pocket payments by the sick or their families at the point of service” [1, p. 24].

In Africa, where states lack either the will or the capacity to apply tax revenues to counter the exclusion caused by user fees, there are two broad alternatives to user fees at the local level. One alternative is to exempt from payment those who are permanently excluded from health care because they are too poor. The other is pre-payment schemes, where people are asked to pay before they need services. Community-based health insurance (CBHI) systems can be considered as one of these pre-payment modalities. In a randomised controlled trial in this issue of *PLoS Medicine*, Evelyn Ansah and colleagues examine the effects of free access to service through pre-payment schemes [3]. Their study is timely, since most international funding agencies seem prepared to support African states that remove user fees.

## What the New Study Adds

Ansah and colleagues' study did not examine wide-scale national experiences of abolishing user fees, as happened in countries such as Niger and Uganda. Rather, the study was a pilot project on free access to a prepayment scheme in the Dangme West District in southern Ghana. In the trial, 2,194 households containing 2,592 Ghanaian children under five years old were randomised into a pre-payment scheme allowing free primary care, or into a control group whose families paid user fees for health care (normal practice). The study also included an observational arm made up of 165 children whose families had previously paid to enrol in the pre-payment scheme. The primary outcome was moderate anaemia (haemoglobin [Hb] < 8 g/dl); secondary outcomes were health care utilisation, severe anaemia, and mortality.

**Linked Research Article**

This Perspective discusses the following new study published in *PLoS Medicine*:

Ansah EK, Narh-Bana S, Asiamah S, Dzordzordzi V, Biantey K, et al. (2009) Effect of removing direct payment for health care on utilisation and health outcomes in Ghanaian children: A randomised controlled trial. PLoS Med 6(1): e1000007. doi: 10.1371/journal.pmed.1000007

Evelyn Ansah and colleagues report on whether removing user fees has an impact on health care-seeking behavior and health outcomes in households with children in Ghana.

Moderate anaemia was detected in 37 children (3.1%) in the control arm and 36 children (3.2%) in the intervention arm (adjusted odds ratio 1.05, 95% confidence interval [CI] 0.66–1.67). There were four deaths in the control and five in the intervention group. Mean Hb concentration, severe anaemia, parasite prevalence, and anthropometric measurements were similar in each group.

This study is important because we still lack knowledge about how this type of pre-payment scheme could be pro-poor. In Rwanda, funding agencies sponsor free CBHI coverage for the worst-off, but research results are not yet available. In fact, it is well known that CBHI [4,5] and cost-recovery schemes based on user fees [6] are not concerned with the worst-off nor with equity.

The purpose of Ansah and colleagues' study was to verify whether free access through pre-payment schemes would improve members' health. Thus, what was being tested was the effectiveness of pre-payment schemes, more than the abolition of user fees. But CBHI effectiveness is not based just on fees but also on the level of trust members have for schemes and health workers, as well as the quality of care, both of which were at the core of the experience. In fact, free access is not enough to ensure that services are used when needed.

This study adds new evidence on pre-payment. It confirms that user fees are only one part of the expenses incurred by the sick. Abolishing fees is therefore not enough to relieve the financial burden, since indirect costs can sometimes be oppressive. The study also showed that pre-payment schemes are not pro-poor, because the worst-off are rarely enrolled. The trial found that membership in a pre-payment scheme leads to greater service utilisation, although the effect was only modest. Children were taken to primary care facilities significantly more frequently in the intervention arm (2.8 episodes per person/year) than in the control arm (2.5 episodes per person/year), but the rate ratio was only 1.12 (95% CI, 1.04–1.20). Health care utilisation in the intervention group (2.8 episodes per person/year) was substantially lower than in the observational arm that self-enrolled in the pre-payment scheme (4.3 episodes per person/year), giving a rate ratio of 0.56 (95% CI, 0.58–0.73). Likewise, enrolment encouraged greater utilisation of formal primary care only among the richest, with no effect on others—suggesting that pre-payment schemes may actually increase inequities.

In sum, this study adds to the current evidence on the limits of local health insurance systems in Africa, where the penetration rate, after more than 15 years of promotion by their organisations, remains very low (5%).

## Methodological Issues in Evaluating Pre-Payment Schemes

The main strength of Ansah and colleagues' new study is in the choice of an experimental design to assess the impact of pre-payment schemes. There is, in fact, a considerable gap between the enthusiasm generated by pre-payment schemes and the scientific evidence to support their use. Most of the published evaluations are based on observational studies that are not very robust [4]. We are aware of only three studies to date that are based on sound experimental designs [7–9].

In addition, the authors are to be congratulated for having decided to assess the intervention's success on the basis of its impacts on health. The evaluation of alternative financing models is too often based on process or output indicators that do not tell us much about the real benefits to populations. Indeed, it is highly questionable to invest important resources in promoting alternative financing models in low- and middle-income countries without convincing evidence of their effectiveness.

A fundamental rule in programme evaluation is that “impact questions should ask whether a program achieved its ultimate objectives” [5]. A pre-payment scheme does aim to increase utilisation and, ultimately, help restore the health of its users. However, these are not the only objectives motivating promoters and members of such schemes. A fundamental function of any health insurance system is to offer effective financial protection to its members, safeguard their assets, and help them escape the medical poverty trap, i.e., the slide into poverty due to costs incurred and income lost because of illness [10]. Health insurance also contributes to the social objective of reducing health care inequities, especially those related to access to services and the burden of illness [11]. Therefore, while it is undoubtedly legitimate to assess a prepayment scheme by considering its impacts on members' health, judging its success solely on such outcomes is inappropriate and possibly misleading.

The main weakness of Ansah and colleagues' study is the way in which the authors assessed the success of the intervention. Several biases have led the authors to judge its success on a very limited basis: (1) although the scheme benefits all members of participating households, the study only took into account a sub-population of beneficiaries (children); (2) in this sub-population, only health-related impacts were considered, and among all possible health benefits, only the potential gains in malaria-related outcomes were considered; and (3) among malaria-related outcomes, the analysis was restricted solely to one indicator: the prevalence of severe and moderate anaemia.

Since the statistical power of the study was limited by the low prevalence of anaemia among the children in the control group (3.1%), and the attributable risk for anaemia of malaria is not known, this study may have been under-powered to detect an effect from the intervention. Even supposing that the intervention did not have the desired effects on malaria-related outcomes, it is still possible that the intervention was associated with improvement in other aspects of children's health. Also, the scheme might have positively affected the health of other groups of enrolees, or provided members with effective financial protection. And we ultimately do not know whether the scheme had favourable distributional (equitable) impacts across social groups.

The study's authors conclude: “This lack of any effect, including on secondary outcomes such as Hb for which the study had good power, challenges the assumption that where introducing free health care leads to changes in utilisation, it can safely be assumed to translate into health benefits. Given the potential size of resources involved in providing free health care that could be diverted from other priorities on the basis of that assumption, this finding is potentially important for policymakers.” [3]. But given the methodological limitations of the study, we believe that the trial provides no scientific evidence on the effectiveness of the pre-payment scheme. It is not correct to conclude, as the authors do, that there is a “lack of any effect” of the intervention. Therefore, we do not think there is any support here for questioning the opportunity to invest in health insurance schemes. We believe that as long as there is no evidence that health insurance schemes are ineffective, protecting families against catastrophic health care costs and removing financial barriers to health care should be a health system priority.

## Implications for Research

In a context of scarce resources, it is essential that interventions be chosen based on conclusive evidence and that outcome evaluations be based on robust designs. But the evaluation of a complex programme such as a prepayment scheme, which has multiple objectives and consequences, cannot be based on an analysis limited to one main outcome. Rather, it requires mobilisation of an array of indicators that can elucidate this complexity and the different causal pathways it puts into play [12]. In that case, a description of the intervention's theory is indispensable [13]. The contribution of qualitative analyses [14], or of evaluation designs that also take the intervention's context into consideration, should also not be overlooked [15,16]. Finally, it is imperative that outcome evaluation be combined with process evaluation. This allows us, particularly, to assess any implementation deficits (type III evaluation errors) [17].

## Equitable Access to Care

Ansah and colleagues' study and the emerging literature on the effects of abolishing user fees in Africa [18] show that lowering financial barriers could promote utilisation of health services, as claimed by the WHO Commission on the Social Determinants of Health [19]. But the decision to abolish fees is not enough. People's trust in their health care services must be restored, and investments (such as salaries and drugs) must be made to improve the service offered. While it is clear fees must be abolished, how to accomplish this is not really known. It is also urgent to evaluate processes, unintended effects, and the actions of those involved in implementation [20,21].

Ultimately, the relationship between abolishing user fees and current health care financing systems in Africa must be closely studied. In Ghana, the nationwide abolition of fees for childbirth services, instituted in 2005, was stopped in 2008 when the state decided to organise a national social insurance system. Moreover, abolition of fees threatens the (rather ineffective) promotion of CBHIs and the sustainability of community-based financing systems. African public health officials and decision makers are worried about the relationship between abolishing user fees and health care financing, and much remains to be done to provide them with the evidence they require.

## Abbreviations

CBHI: community-based health insurance
CI: confidence interval
Hb: haemoglobin
WHO: World Health Organization
